# Do Robotics and Virtual Reality Add Real Progress to Mirror Therapy Rehabilitation? A Scoping Review

**DOI:** 10.1155/2018/6412318

**Published:** 2018-08-19

**Authors:** Nelly Darbois, Albin Guillaud, Nicolas Pinsault

**Affiliations:** ^1^Critical Thinking Research Federation FED 4276, University Grenoble-Alpes, Grenoble, France; ^2^Cortecs team, Grenoble, France; ^3^School of Physiotherapy, Grenoble-Alpes University Hospital, Grenoble, France; ^4^ThEMAS team, TIMC-IMAG Laboratory, UMR CNRS-UGA 5525, Grenoble, France

## Abstract

**Background:**

Mirror therapy has been used in rehabilitation for multiple indications since the 1990s. Current evidence supports some of these indications, particularly for cerebrovascular accidents in adults and cerebral palsy in children. Since 2000s, computerized or robotic mirror therapy has been developed and marketed.

**Objectives:**

To map the extent, nature, and rationale of research activity in robotic or computerized mirror therapy and the type of evidence available for any indication. To investigate the relevance of conducting a systematic review and meta-analysis on these therapies.

**Method:**

Systematic scoping review. Searches were conducted (up to May 2018) in the* Cochrane Library, Google Scholar, IEEE Xplore, Medline, Physiotherapy Evidence Database*, and* PsycINFO* databases. References from identified studies were examined.

**Results:**

In sum, 75 articles met the inclusion criteria. Most studies were publicly funded (57% of studies; n = 43), without disclosure of conflict of interest (59% of studies; n = 44). The main outcomes assessed were pain, satisfaction on the device, and body function and activity, mainly for stroke and amputees patients and healthy participants. Most design studies were case reports (67% of studies; n = 50), with only 12 randomized controlled trials with 5 comparing standard mirror therapy versus virtual mirror therapy, 5 comparing second-generation mirror therapy versus conventional rehabilitation, and 2 comparing other interventions.

**Conclusion:**

Much of the research on second-generation mirror therapy is of very low quality. Evidence-based rationale to conduct such studies is missing. It is not relevant to recommend investment by rehabilitation professionals and institutions in such devices.

## 1. Introduction

Mirror therapy was originally described by Ramachandran and Rogers-Ramachandran, who suggested its use in amputees with phantom limb pain [1]. They introduce an inexpensive new device: a mirror was placed vertically on a table so that the mirror reflection of the patient's intact hand was superimposed on the felt position of the phantom [1]. This standard mirror therapy has been used in rehabilitation for multiple indications since the 1990s [2]. A good level of evidence supports some of these indications, particularly for cerebrovascular accidents in adults [3, 4] and cerebral palsy in children [5]. Cost is very low, because a simple little and not specifically dedicated mirror can be used [1]. Dedicated mirror boxes cost about $65 each [6].

Since 2000s, virtual reality or robot has been developed and marketed to treat various diseases as a more technologically sophisticated version of the standard mirror therapy introduced in 1996 [7, 8]. Robotic devices and virtual reality are increasingly used and assessed in rehabilitation and research [9, 10]. This second-generation devices are probably much more expensive than standard mirror therapy: they often present a technological complexity that requires investment, constant maintenance, and highly qualified operators [11]. Low cost virtual reality device costs about $252 to purchase [12]. Low cost robotic device for robotic gait rehabilitation was estimated to cost $25,000, which is less than 10% of the price of device currently available in Brazil for the same indication [13]. For some indications, virtual reality such as robotics has no greater effectiveness than more conventional techniques [14, 15]. Studies evaluating the impact on various outcomes of these mirror therapy devices exist [8, 16, 17], but no review summarizes the available data.

The purpose of this review was as follows: (1) to map the extent, nature, and rationale of research activity in robotic or computerized mirror therapy; (2) to summarize the main sources and types of evidence available about the effectiveness of these therapies for any indication; (3) to investigate the relevance of conducting a systematic review and meta-analysis on these therapies.

## 2. Methods

Systematic scoping review was conducted. The methods are based on literature-based guidelines [18, 19].

### 2.1. Search Strategy

An extensive search of the published and grey literature was conducted. The following electronic databases were searched for articles published in 1996 up to May 2018:* Cochrane Library, Google Scholar, IEEE Xplore, Medline, Physiotherapy Evidence Database (PEDRO)*, and* PsycINFO.* The search combined terms for mirror therapy and computerized or robotic system. More details on the search strategies used within each database are in Table 1. In addition, the references lists of included studies were reviewed (complementary strategy).

### 2.2. Eligibility Criteria

The inclusion criteria were as follows:

- Type of study design: randomized controlled trials (RCTs), nonrandomized controlled trials (NRCTs), interrupted time series (ITS), before-after studies (controlled or not), cohort studies, case-control studies, cases series or case reports, systematic or scoping review, and meta-analysis.
- Type of intervention: computerized or robotic based on mirror therapy or full body illusion (with or without additional techniques).
- Type of participants: healthy subjects or any kind of patients.
- Type of control: none of any kind of control.
- Type of outcomes: any kind of outcomes.
- Languages: English, Esperanto, French, German, Italian, Portuguese, and Spanish.

The exclusion criteria were as follows:

- Type of study design: feasibility study not on patients or healthy volunteers, technical or development description, protocol, expert opinion, and personal observation.
- Type of intervention: transcranial stimulation, electrostimulation, real mirror, or standard mirror box (without computerized or robotic mirror therapy), and computerized or robotic therapy not based on mirror therapy or full body illusion.
- Type of participants: nonhuman animal.
- Accessibility: only an abstract being available.
- Originality: data and method not original, already included in the review.

### 2.3. Study Selection

First, the selection was made by title. Secondly, the abstracts of each study were analysed. Studies that did not meet the eligibility criteria on the basis of the content of their abstracts were excluded. Full-texts of the remaining studies were obtained and the eligibility criteria were again applied.

For references obtained with the complementary approach, the study abstracts were analysed. If required, the full-text versions were obtained to determine whether the studies met our eligibility criteria.

### 2.4. Level of Evidence

The methodological quality or risk of bias of the included studies has not been appraised. This is consistent with guidance on scoping review conduct [18, 19]. Only the general level of evidence has been specified, according to* The Oxford 2011 Levels of Evidence* [20]. The general level of evidence for each study was appraised by one reviewer. For RCTs on second-generation mirror therapy versus standard mirror therapy or conventional rehabilitation, the presence or absence of single or double-blindness procedure and primary outcome were noted.

### 2.5. Data Extraction

Data were extracted by one reviewer into standardised and structured tables. The main data extracted were year of publication, continent, funding, conflict of interest disclosure, rationale for conducting the study, design, type of intervention, number, length and frequency of sessions, type and number of participants, type of outcomes, follow-up duration, main findings, side effects, and suggested indications for using computerized or robotized mirror therapy.

### 2.6. Data Synthesis

Flow diagram has been created to show the study selection process. The findings were summarized with a narrative description and tables. Considering the large number of studies included and the research objectives, only RCTs on second-generation mirror therapy versus standard mirror therapy or conventional rehabilitation were presented individually.

## 3. Results

### 3.1. Selection of Studies

**Figure 1:**
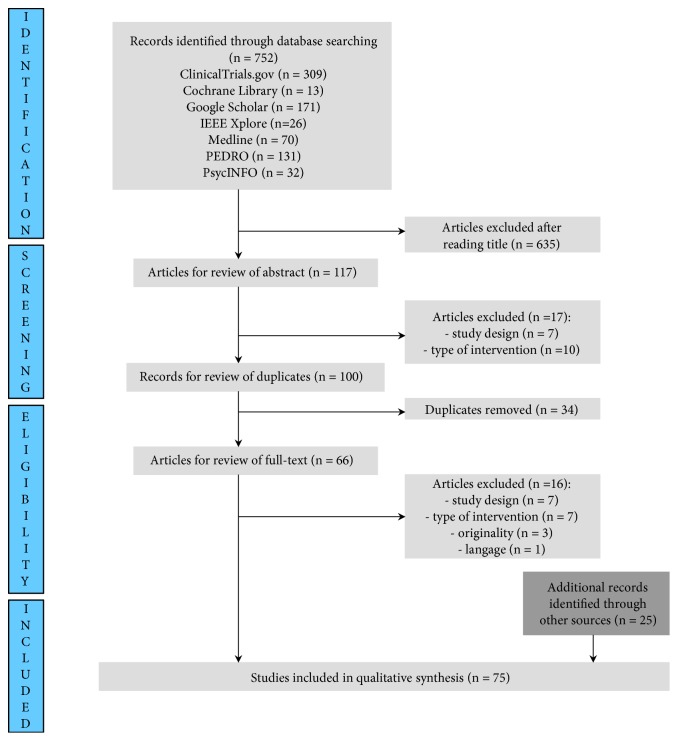
Flow chart of the study selection process.

Of the 752 article titles identified by the standard search procedure, 50 articles met the inclusion criteria [12, 16, 21–69]. The complementary search strategy gave 25 more articles [7, 8, 17, 70–91]. Reasons for exclusion after reading the abstract or the full-text were the following: type of study design (n = 14), type of intervention (n = 17), originality (n = 3), or language (n = 1).  Figure 1 shows flow diagram of the study selection process.

### 3.2. General Characteristics of Included Studies

The first paper was published in 2002 [7]. Sixteen percent of the papers (n = 12) were published in the 2000s and 84% (n = 86) in the 2010s (Table 2).

The studies were conducted in Europe (n = 29), Asia (n = 23), North America (n = 19), and Australia or New Zealand (n = 8) (Table 3).

Most studies were publicly sponsored (57% of studies; n = 43), 9 were privately sponsored, 2 were not sponsored, and for 21 studies funding sources were not reported (Table 4).

A disclosure of conflict of interest was missing from 59% of the studies (n = 44). For those who reported them, 72% (n = 18) declare that the research was conducted in the absence of any commercial or financial relationships that could be construed as a potential conflict of interest, and 28% (n = 7) reported disclosures relevant to the manuscript (Table 5).

### 3.3. Rationales of Included Studies

The authors justified the relevance of studying second-generation mirror therapy rather than standard mirror therapy in 65% (n = 49) of the included studies. The three most frequent justifications were as follows: to facilitate neuroplasticity through multisensory feedback (not visual only), to increase the range and difficulty of possible training task, and to stimulate patient motivation and engagement (Table 6). For an exhaustive list of rationales, see Table S1 in Supplementary Materials.

### 3.4. Design of Included Studies

Case series/reports are the most common design (68% of studies; n = 51) (Table 7). Only 12 RCTs and 3 NRCTs are included. In the 12 RCTs included, 5 trials compare standard mirror therapy to virtual mirror therapy [21–24, 78]. The others compare second-generation mirror therapy to conventional rehabilitation [6, 27, 51, 52, 58] or different modalities of computer-based mirror therapy [32, 40].

### 3.5. Type of Intervention

The studies mainly assess the effect of virtual reality (65%; n = 49) [8, 16, 21–23, 28–32, 37–43, 45–48, 50–53, 57–59, 61–65, 67, 69, 72–74, 77–80, 82, 84–89] and robotic system (23%; n= 17) [7, 12, 25, 26, 34, 49, 54–56, 60, 66, 70, 71, 75, 76, 81, 83] (Table 8). The names of the devices evaluated in the different studies are as follows: Bi-Manu-Track, BioPatRec, Dulex-II, HapticMASTER, Mirror Image Movement Enabler (MIME), Neuromotus, Picdae Robot, Pictogram round (Orange Foundation), TheraMem, Trinus Virtual Reality, Virtual Reality-based Mirror Visual Feedback, VR-Mirror (MedICLab), and YouRehab. The names used to designate the different types of second-generation mirror therapy devices are listed exhaustively in Table S3 (in Supplementary Materials).

Additional treatment or device is present in 12 studies: myoelectric control (n = 3), electro-encephalogram (n = 1), transcranial magnetic stimulation (n = 3), force platform (n = 1), tendon vibration (n = 1), functional electrical simulation (n = 1), machine learning (n = 1), and electromyography signal (n = 1).

Most often only one session was performed (40% of studies; n = 30) (Table 9). When several sessions occurred, they were most often conducted 5 times a week (12% of studies; n = 9) and 2 to 4 times a week (11% of studies; n = 8) (Table 10). The duration of the sessions was most often not mentioned (39% of studies; n = 29). When mentioned, it was most often a 30-minute session (12% of studies; n = 9) (Table 11).

Only one study mentioned the cost of the device [12]. The device was described as low cost and cost for parts and materials was about $252. The installation time before each session was given in only 4 studies; it was 3 to 5 minutes.

### 3.6. Type and Number of Participants

Participants in each included study were from 1 to 54 (Table 12). A significant number of studies involved only one participant (15%; n = 11).

Twenty-eight studies were conducted on healthy patients. The three most frequent pathologies in patients included were hemiplegia after stroke (n = 30), amputees with phantom limb pain (n = 18), and complex regional pain syndrome (n = 2) (Table 13).

### 3.7. Type of Outcomes

Forty-five different outcomes are used in all studies. The three most frequently used outcomes are pain, satisfaction with the system, and body functions and activities (Table 14). See Table S2 in Supplementary Materials for an exhaustive list.

Most often these outcomes were assessed over a period of less than 24 hours (43%; n = 32). Seven studies did not report the duration of follow-up (Table 15).

### 3.8. Findings of Included Studies

Most studies found a positive effect after second-generation mirror therapy sessions (such as decreased pain, increased motor skills or satisfaction, or decreased spasticity) for some outcomes or patients (81% of studies; n = 61). Fourteen studies showed a positive effect for all outcomes and patients (19% of studies) (Table 16).

Many studies have not mentioned anything about the recording of possible side effects (84% of studies; n = 55) (Table 17). The side effects identified are as follows: increment in pain for a period, muscle cramp, lack of comfort, and intensification of phantom experience.

### 3.9. Suggested Indications

Various possible indications of second-generation mirror therapy were mentioned by the authors of the included studies. The three most frequent were as follows: stroke patients (55% of studies; n = 41), phantom limb pain (33% of studies; n = 25), and complex regional pain syndrome (8% of studies; n = 6) (Table 18).

### 3.10. Level of Evidence

General level of evidence of included studies was very low (Oxford level of evidence: 2 to 4), with a large majority of 4/5 level studies (1/5 is the best level, 5/5 is the worst) (Table 19).

RCTs on conventional versus second-generation mirror therapy are presented in Table 20. None of them indicate a primary outcome; two are single-blinded, and three were without blindness procedure. RCTs on second-generation mirror therapy versus conventional rehabilitation are presented in Table 21. Two of them indicate a primary outcome; four are single-blinded, and one was without blindness procedure. The others two RCTs compare different virtual reality modalities [82, 90].

## 4. Discussion

### 4.1. Summary of Findings

We have mapped the extent, nature, and rationale of research activity in robotic or computerized mirror therapy. The main sources and types of evidence available about the effectiveness of these therapies for any indication are case series or reports. Only five RCTs on conventional versus second-generation mirror therapy exist, and five on second-generation mirror therapy versus conventional rehabilitation. Owing to the heterogeneity of included studies, a meta-analysis was not considered to be appropriate. There is either an absence of a rationale, or a nonevidence-based rationale to justify the conduct of studies on the efficacy of second-generation mirror therapy, despite public funding. Disclosure of conflict of interest was missing for a majority of the included studies.

### 4.2. Strengths and Weaknesses of the Review

The major strength of this review is the extensive search in 6 electronic databases, especially in the search engine of the world's largest technical professional organization dedicated to advancing technology (*IEEE Xplore*). Moreover, the inclusion criteria were broad: seven languages were accepted, as well as any type of design. A potential limitation is that the search was conducted only in English or French in the electronic databases. However, about 1/3 of the studies included come from the Asian continent. Scientific document in Asian language (and particularly Chinese) being prevalent [92], it is possible that there are other studies of better quality in Asian language. However, we systematically reviewed the references lists of included studies, and we have not identified bibliographic references in Asian language in articles by Asian authors. The large number of different types of device name (see Table S3 in Supplementary Materials) probably explains the high number of studies included thanks to the complementary search. The main search did not take into account all the keywords, but this scoping review allowed identifying these keywords in a more exhaustive way.

### 4.3. Interpretation of Findings

#### 4.3.1. Very Low Level of Evidence

Many studies on second-generation mirror therapy, although publicly funded, have very low levels of evidence. Indeed, internal validity of case series or reports is usually very low, due to the lack of a control group [93]. For example, the effects observed may be wholly or partly due to the placebo effect, research participation effects [94], or the natural history effect [95]. Case series and reports have a great role in pharmacovigilance, rare diseases, or medical education, but not to assess the effectiveness of techniques on relatively common pathologies such as hemiplegia after stroke [96]. Similar limitations exist regarding the internal validity of noncontrol before-after studies.

One explanation for the prevalence of these poor quality studies is the cost and time already invested for the development of the devices [11, 97]. To conduct randomized controlled trials rather than case studies is indeed more costly and time-consuming [98].

#### 4.3.2. Meta-Analysis Not Relevant

If performed, a meta-analysis on second-generation mirror therapy could have as therapeutic control group standard mirror therapy, placebo, or conventional rehabilitation. Ten RCTs meet this requirement (See Tables 20 and 21). However, owing to the heterogeneity of interventions (virtual reality or robot, for arm, leg, or full body), settings (from only one session to 24 sessions during 2 months), participants (healthy participants and various stroke patients), outcome measures (such as perceptions, corticospinal excitability, balance, facial movements, motor impairment), and control groups (real mirror or various rehabilitation training), a meta-analysis is not considered to be appropriate, although it is always difficult deciding just how similar they need to be [99]. This is compounded by the risk of bias in these studies: only 2 have defined primary outcome, and 3 have no blindness procedures.

Even for the most studied population (hemiplegia after cerebral stroke), a meta-analysis does not seem relevant: 7 RCTs are interested in this population, but the interventions are different (robotic mirror therapy (n=4) and virtual reality mirror therapy (n=4)), as are the outcomes investigated (general motor function (n=4), corticospinal excitability (n=1), pusher syndrome (n=1), balance (n=1), lower extremity motor function (n=1), lower limb function (n=1), reach extent (n=1), measure of abnormal synergies (n=1), hemiplegic arm activity (n=1), bilateral arm coordination (n=1), facial movement (=1)), and the number of sessions (9 to 30). The only corpus of studies sufficiently homogeneous to conduct a meta-analysis would be that constituted by the studies of Lum et al. 2002 [7] and 2006 [25], Burgar et al. 2011 [26], and Liao et al. 2011 [81]. However, they include only 131 patients in total. Moreover, the risk of bias in these studies is probably high (simple blinding procedure and no primary outcome for 2 studies). Consequently, conducting a meta-analysis is not relevant.

Batson et al. [100] assessed the quality of evidence used in manufacturers' submissions for technology appraisal in United Kingdom. It is an important factor in receiving a positive recommendation to recommend the use of new technology in guidelines by National Institute for Health and Care Excellence. They mention the frequent risks of bias in included studies and the failure to explore heterogeneity.

#### 4.3.3. No Evidence-Based Rationales

Rationales to conduct studies on computerized or robotic mirror therapy rather than standard mirror therapy often mention the possibility of intensive and repetitive training and a better or faster recovery (see Results/Rationale of included studies). However, these arguments are not evidence-based. In the present included studies, computerized or robotic sessions occurred mainly 5/weeks during 30 minutes (see Results/Type of intervention). However, standard mirror therapy sessions are also frequent [4, 5]. In addition, the greater effect of robotic or virtual reality rehabilitation rather than conventional rehabilitation is not sure. Cochrane systematic reviews on this topic concluded that “*virtual reality and physiotherapy may have similar effects on gait, balance, and quality of life*” for Parkinson disease [14] or that “*the use of virtual reality and interactive video gaming was not more beneficial than conventional therapy approaches in improving upper limb function*” after stroke [101]. When there is effect of robot-assisted training, the quality of evidence is very low or low for improving arm functions after stroke [102] or moderate for improving independent walking in people after stroke [103]. Researchers who have conducted systematic reviews on the topic suggest that the effectiveness of robot-assisted therapy is more due to the high intensity of training than to the treatment modality [102–106]: “*it seems unlikely that therapy provided by robots will lead to better results than therapy provided by humans under the premise that intensity, amount and frequency of therapy are exactly comparable*” [104]. In the specific field of robotic mirror therapy, Burgar et al. give weight to this hypothesis [26].

The absence of a rationale or evidence-based rationale to justify the conduct of studies on the efficacy of second-generation mirror therapy can be explained in several ways. First, researchers would not be trained and encouraged enough to justify the relevance of their research. Therefore, funders may not have sufficient trained staff to rigorously assess the rationale for research projects. Bujar et al. show that quality process during drug development, regulatory review, and health technology assessment is poor, because there is limited training in the science of decision-making from pharmaceutical companies and regulatory authorities [107]. Intellectual bias occur during meeting, which may lead the committee members to believing information which appears more favourable or familiar [108]. Secondly, the commercial interests can take precedence over the public health justification of investing money and time in research in this field. Gøtzsche argues that research ethics committees should require a systematic review of similar previous trials in the application to allow a study to be carried out, so that economic interests do not outweigh the social benefits [109].

#### 4.3.4. Conflict of Interest

Conflict of interest statements may temper the enthusiasm for dataregarding a new device because of the risk of bias of the investigator. Indeed, “*sponsorship of drug and device studies by the manufacturing company leads to more favourable efficacy results and conclusions than sponsorship by other sources*” [110]. In the included studies in this review, disclosure of conflict of interest was missing from 59% of the studies, and funding sources were not reported for 28%. For those who reported them, 28% reported disclosures relevant to the manuscript, and 12% were privately sponsored. But undeclared payments or funding may occur. Patel et al. show that it was common for payments from Intuitive (the manufacturer of the Da Vinci Robotic Surgery system) to be undeclared in robotic surgery articles [111].

#### 4.3.5. Choice of Control Group Intervention

Among the 10 randomized controlled trials evaluating the efficacy of the second-generation mirror therapy, 5 have as control group conventional rehabilitation and not first-generation mirror therapy.

However, there is evidence of the effectiveness of first-generation mirror therapy for some indications [3–5]. Second-generation therapies are also more expensive. With the purchase, a mirror box costs about $65 [6], and it is possible to manufacture one so that it is less expensive. Second-generation low cost installations cost at least $250 [12] and $25,000 [13], respectively (ritual virtuality and robotics). The costs of maintenance and professional training to learn how to use the devices must be added.

If the efficiency of second-generation devices is not higher than that of first-generation devices, they are therefore not to be preferred given their costs. Only trials comparing second-generation therapy to first-generation therapy can clarify this point.

### 4.4. Implications for Practice, Research, and Policy

Given the absence of good empirical evidence of second-generation mirror therapy efficiency, it is not relevant to recommend investment by rehabilitation professionals and institutions in such devices. The practice of mirror therapy with a real mirror, less costly to acquisition, maintenance, and training should be preferred.

Investigators should no longer conduct any more case or report studies on computerized or robotic mirror therapy. They should conduct randomized controlled trials, registered on* clinicaltrial.org* to limit publication bias. Investigators should use first-generation mirror therapy as control group and systematic and comprehensive disclosure of funding and conflicts of interest. It does not seem reasonable to develop new devices given the costs, time, and resources required, but rather to assess existing devices, especially in the case of public funding. On the other hand, research on mirror therapy with a real mirror or mirror box should continue to emerge given the good quality of evidence already available for certain indications [3–5]. If a new literature review is conducted on second-generation mirror therapy, search engine queries to identify studies should use comprehensive keywords (see Table S3 in Supplementary Materials).

Public funders and research ethics committees should require evidence-based rationale (with, for example, systematic review of similar previous trials) for ethics of funding approval.

### 4.5. Conclusion

Despite public funding, much of the research devoted to second-generation mirror therapy is of very low quality. The main sources and types of evidence available about the effectiveness of these therapies for any indication are case series or reports. Only five RCTs on conventional versus second-generation mirror therapy exist and five on second-generation mirror therapy versus conventional rehabilitation. Evidence-based rationale to conduct such studies is often missing. It is not relevant to recommend investment by rehabilitation professionals and institutions in such devices. It does not seem reasonable to develop new devices given the costs, time, and resources required, but rather to assess existing devices with well-conducted randomized controlled trials, especially in the case of public funding.

## Figures and Tables

**Table 1:** Full search strategy.

Database	Term(s) (entered in the basic search bar)
*Cochrane Library*	mirror

*Google Scholar*	“mirror therapy” or “mirror visual feedback” or “mirror box” or “mirror reflection”

*IEEE Xplore*	“mirror therapy”

Medline	(computer [tiab] or computerised [tiab] or computerized [tiab] or technology [tiab] or “tablet PC” [tiab] or “machine learning” [tiab] or augmented [tiab] or virtual [tiab] or robotic [tiab] or robotics [tiab] or exoskeleton [tiab] or robot [tiab] or “video games” [mesh] or “virtual reality” [mesh] or “Virtual Reality Exposure Therapy” [mesh] or robotics [mesh] or “Exoskeleton Device” [mesh] or “Therapy, Computer-Assisted” [mesh] or “artificial intelligence” [tiab] or “Brain-computer interfaces” [mesh]) and (“mirror therapy” [tiab] or “mirror visual feedback” [tiab] or “mirror box” [tiab] or “mirror reflection” [tiab])

*PEDRO*	mirror (in title or abstract)

*PsycINFO*	(TI computer or AB computer or TI computerised or AB computerised or TI computerized or AB computerized or TI technology or AB technology or TI “tablet PC” or AB “tablet PC” or TI “machine learning” or AB “machine learning” or AB augmented or TI augmented or TI virtual or AB virtual or AB robotic or TI virtual or TI robotics or AB robotics or TI exoskeleton or AB exoskeleton or TI robot or AB robot or TI “artificial intelligence” or AB “artificial intelligence” or MA “Computer Games” or MA “virtual reality” or MA robotics) and (AB “mirror therapy” or TI “mirror therapy” or AB “mirror visual feedback” or TI “mirror visual feedback” or AB “mirror box” or TI “mirror box” or AB “mirror reflection” or TI “mirror reflection” or MA “mirror image”)

**Table 2:** Years of publication of included studies.

**Year**	**Number of studies**
2002-2006	8

2007-2011	10

2012-2016	37

2017-2018 (May)	20

**Table 3:** Continents of included studies.

**Continent**	**Number of studies**
Europe	29

Asia	23

North America	19

Australia and New Zealand	8

Africa	0

Central and South America	0

**Table 4:** Funding of included studies.

**Funding**	**Number of studies**
Publicly sponsored	43

Privately sponsored	9

Not sponsored	2

Not reported	21

**Table 5:** Disclosure of conflict of interests in included studies.

**Conflict of interest disclosure**	**Number of studies**
Missing disclosure	49

Absence of conflict interest	19

Presence of conflict interest	7

**Table 6:** Rationale cited in the studies for conducting research on mirror robotic systems or mirror virtual reality rather than standard mirror therapy.

**Rationale**	**Number of studies**
Multisensory feedback (to facilitate neuroplasticity)	19

To increase the range and difficulty of possible training task	15

To increase motivation and engagement	11

Intensive and repetitive training	9

Customizable environments	9

To accomplish different bimanual coordination movements	7

Faster or greater recovery	7

**Table 7:** Design of included studies.

**Design**	**Number of studies**
Case series/reports	50

RCT	12

Non-controlled before-after study	8

NRCT	3

ITS	1

Review	1

**Table 8:** Type of intervention of included studies.

**Intervention**	**Number of studies**
Virtual reality	49

Robotic	17

Robotic and virtual reality	4

Video	2

Tablet-PC	1

Video and virtual reality	1

Medical ultrasound imaging	1

**Table 9:** Number of sessions in included studies.

**Sessions**	**Number of studies**
1	30

5 to 10	15

11 to 20	11

2 to 4	10

Unknown	4

20 to 30	3

>30	2

**Table 10:** Session frequency in included studies.

**Sessions**	**Number of studies**
1 only session	30

5/week	9

2 to 4/week	8

Unknown	7

1 to 2/week	4

<1/week	2

3 to 5/week	2

1/week	1

1 to 2/day	1

**Table 11:** Session length in included studies.

**Session length**	**Number of studies**
Unknown	29

30 min	9

1 hour	7

45 min	6

15 min	5

20 min	4

10 min	2

60 to 90 min	2

No time limit	1

25 to 60 min	1

90 to 105 min	1

**Table 12:** Number of participants in each included study.

**Number of participants**	**Number of studies**
1	11

2 to 9	29

10 to 19	14

20 to 30	16

31 to 54	3

Unknown	2

**Table 13:** Health status of participants in included studies.

**Participants**	**Number of studies**
Hemiplegia after stroke	30

Healthy	28

Amputees with phantom limb pain	18

Complex regional pain syndrome	2

Neuropathic pain	2

Autism spectrum disorder	1

Hand-injury	1

Pusher syndrome after stroke	1

Spinal cord injury	1

Stroke patient with central facial paresis	1

**Table 14:** The most frequently used outcomes in included studies.

**Outcomes**	**Number of studies**
Pain	25

Satisfaction with the device	18

Body functions and activities	17

Motor assessment	10

Spasticity level	9

Illusion intensity	8

**Table 15:** Follow-up period in included studies.

**Follow-up period**	**Number of studies**
< 24 hours	32

2 to 7 days	4

2 to 6 weeks	19

2 months	4

3 months	4

6 months	4

2 years	1

Unknown	7

**Table 16:** Positive effect in included studies.

**Positive effect**	**Number of studies**
On some assessed outcomes or patients	61

On all assessed outcomes and patients	14

**Table 17:** Side effects in included studies.

**Side effects**	**Number of studies**
Therapists or patients could report any adverse event	12
(i) no adverse effect	5
(ii) adverse effect	7

Not mentioned	63

**Table 18:** Suggested indications for the use of second-generation mirror therapy in included studies (the study was not necessarily conducted on this type of population).

**Indications**	**Number of studies**
Stroke patients	41

Phantom limb pain	25

Complex regional pain syndrome	6

Chronic pain management	5

Rehabilitation of motor function	3

Cerebral palsy	2

Autism spectrum disorders	1

Cerebral ataxia	1

Fibromyalgia	1

Fracture	1

Hand injury	1

Motion analysis	1

Other neuropathic pain	1

Pusher syndrome	1

Spinal cord injury	1

**Table 19:** Level of evidence according to *The Oxford 2011 Levels of Evidence* [20].

**Design**	**Number of studies**	**Level of evidence**
Case series/reports	50	4/5

RCT	12	2/5
(i) first VS second generation	5	2/5
(ii) second generation mirror therapy VS conventional rehabilitation	5	2/5
(iii) others	2	2/5

Non-controlled before-after study	8	4/5

NRCT	3	3/5

ITS	1	3/5

Review	1	-

**Table 20:** Characteristics of included RCTs (n = 5) which compare first- and second-generation mirror therapy.

**Source**	**Participants **	**Intervention**	**Comparator**	**Sessions**	**Primary outcome**	**Blinding**	**Main findings**
Regenbrecht et al., 2011 [78]	24 healthy subjects	Augmented mirror box (AMB)	Optical mirror box	1	No	No blinding	The mirror box technique is able to fool or confuse individual's perceptions and beliefs. The AMB produced strong results in this regard.

Hoermann et al., 2012 [24]	21 healthy subjects	Video-mediated (advanced) augmented reflection technology	Optical mirror box	1	No	No blinding	Video-mediated manipulations of hand-position reversals produced equal to stronger effects of ownership compared with the mirror reflection.

Kang et al., 2012 [21]	18 healthy subjects and 18 hemiplegic patients	Virtual mirror therapy	Relaxation or real mirror	1	No	No blinding	Corticospinal excitability was facilitated to a greater extent in the virtual mirror paradigm than in the real mirror.

Yang et al., 2014 [22]	12 stroke patients with pusher syndrome	Computer-generated visual feedback training	Mirror visual feedback training	3 times a week during 3 weeks	No	Simple blinding (assessors)	The computer-generated visual feedback training more effectively aided recovery from pusher syndrome and balance (but no significant difference was noted between groups for lower extremity motor function).

In et al., 2016 [23]	25 patients with chronic stroke	Virtual reality reflection therapy (VRRT)	Standard mirror therapy	5 time a week during 4 weeks	No	Simple blinding (assessors)	Applying VRRT might be even more beneficial than conventional rehabilitation program alone in improving affected lower limb function.

**Table 21:** Characteristics of included RCTs (n = 5) which compare second-generation mirror therapy and conventional rehabilitation.

**Source**	**Participants**	**Intervention**	**Comparator**	**Sessions**	**Primary outcome**	**Blinding**	**Main findings**
Lum et al., 2002 [7]	27 patients with chronic hemiparesis	Robotic mirror therapy	Neurodevelopmental therapy	24 sessions during 2 months	No	Simple blinding (outcome raters)	The robot group had larger improvements in a portion of the Fugl-Meyer test after 1 and 2 months of treatment, in strength and larger increases in reach extent after 2 months. At the 6-month follow-up, the groups no longer differed in terms of the Fugl-Meyer test; however, the robot group had larger improvements in the FIM™.

Lum et al., 2006 [25]	30 subacute stroke patients	Robot-assisted treatment (unilateral, bilateral or combined)	Neurodevelopmental therapy	15 sessions during 4 weeks	No	Simple blinding (outcome raters)	Robotic training compared with conventional therapy produced larger improvements on a motor impairment scale and a measure of abnormal synergies. However, gains in all treatment groups were equivalent at the 6-month follow-up.

Burgar et al., 2011 [26]	54 hemiparetic patients	Usual care and robot-assisted therapy (low or high dose)	Usual care and additional conventional therapy	15 to 30 sessions during 3 weeks	Fugl-Meyer Assessment	Simple blinding (outcome raters)	Gains in the primary outcome measure were not significantly different between groups at follow-up.

Liao et al., 2011 [81]	20 post stroke patients	Robot-assisted therapy	Dose-matched active control therapy	20 sessions during 4 weeks	Ratio of mean activity between the impaired and unimpaired arm	Simple blinding (outcome rater)	The robot-assisted therapy group significantly increased motor function, hemiplegic arm activity and bilateral arm coordination compared with the dose-matched active control group.

Kang et al., 2017 [27]	21 post stroke patients with central facial paresis	Orofacial exercise and mirror therapy using a tablet PC	Orofacial exercise	Twice daily for 14 days	No	No blinding	The degree of improvement of facial movement was significantly larger in the mirror group than in the control group.

## Supplementary Materials

Supplementary Materials Table S1. Exhaustive list of rationales cited in the studies for conducting research on mirror robotic systems or mirror virtual reality rather than standard mirror therapy. Table S2. Exhaustive list of outcomes nature used in the included studies. Table S3. Exhaustive list of names used in the included studies to designate the different types of second-generation mirror therapy device.

## References

[1] Ramachandran V. S., Rodgers-Ramachandran D. (1996). Synaesthesia in phantom limbs induced with mirrors. *Proceedings of the Royal Society B Biological Science*.

[2] Deconinck F. J., Smorenburg A. R., Benham A., Ledebt A., Feltham M. G., Savelsbergh G. J. (2015). Reflections on mirror therapy: a systematic review of the effect of mirror visual feedback on the brain. *Neurorehabilitation and Neural Repair*.

[3] Broderick P., Horgan F., Blake C., Ehrensberger M., Simpson D., Monaghan K. (2018). Mirror therapy for improving lower limb motor function and mobility after stroke: A systematic review and meta-analysis. *Gait & Posture*.

[4] Thieme H., Mehrholz J., Pohl M., Behrens J., Dohle C. (2012). Mirror therapy for improving motor function after stroke.. *Cochrane Database of Systematic Reviews (Online)*.

[5] Park E.-J., Baek S.-H., Park S. (2016). Systematic review of the effects of mirror therapy in children with cerebral palsy. *Journal of Physical Therapy Science*.

[6] Mirror Box. https://www.edgemobilitysystem.com/products/mirror-box.

[7] Lum P. S., Burgar C. G., Shor P. C., Majmundar M., Van der Loos M. (2002). Robot-assisted movement training compared with conventional therapy techniques for the rehabilitation of upper-limb motor function after stroke. *Archives of Physical Medicine and Rehabilitation*.

[8] Gaggioli A., Morganti F., Meneghini A. (2005). The virtual reality mirror: mental practice with augmented reality for post-stroke rehabilitation. *Annual Review of CyberTherapy and Telemedicine*.

[9] Krebs H. I., Volpe B. T. (2013). Rehabilitation robotics. *Handbook of Clinical Neurology*.

[10] Cano Porras D., Siemonsma P., Inzelberg R., Zeilig G., Plotnik M. (2018). Advantages of virtual reality in the rehabilitation of balance and gait. *Neurology*.

[11] Keller T., Perry J. (2018). *Rehabilitation Robotics: From Expensive Tools for Specialized Hospitals towards Home and Tele-Rehabilitation Use*.

[12] Morris C., Fu Y., McCormick S., Wachter B., Devasia S. Low-cost assistive robot for mirror therapy rehabilitation.

[13] Volpini M., Bartenbach V., Pinotti M., Riener R. (2017). Clinical evaluation of a low-cost robot for use in physiotherapy and gait training. *Journal of Rehabilitation and Assistive Technologies Engineering*.

[14] Dockx K., Bekkers E. M. J., Van den Bergh V. (2016). Virtual reality for rehabilitation in Parkinson disease. *The Cochrane Library*.

[15] Labruyère R., Van Hedel H. J. A. (2014). Strength training versus robot-assisted gait training after incomplete spinal cord injury: A randomized pilot study in patients depending on walking assistance. *Journal of NeuroEngineering and Rehabilitation*.

[16] Hoermann S., Ferreira dos Santos L., Morkisch N. (2017). Computerised mirror therapy with Augmented Reflection Technology for early stroke rehabilitation: clinical feasibility and integration as an adjunct therapy. *Disability and Rehabilitation*.

[17] Ortiz-Catalan M., Guðmundsdóttir R. A., Kristoffersen M. B. (2016). Phantom motor execution facilitated by machine learning and augmented reality as treatment for phantom limb pain: a single group, clinical trial in patients with chronic intractable phantom limb pain. *The Lancet*.

[18] Peters M. D. J., Godfrey C. M., Khalil H., McInerney P., Parker D., Soares C. B. (2015). Guidance for conducting systematic scoping reviews. *International Journal of Evidence-Based Healthcare*.

[19] Tricco A. C., Lillie E., Zarin W. (2016). A scoping review on the conduct and reporting of scoping reviews. *BMC Medical Research Methodology*.

[20] OCEBM-Levels of Evidence Working Group (2011). *The Oxford 2011 Levels of Evidence*.

[21] Kang Y. J., Park H. K., Kim H. J. (2012). Upper extremity rehabilitation of stroke: facilitation of corticospinal excitability using virtual mirror paradigm. *Journal of NeuroEngineering and Rehabilitation*.

[22] Yang Y.-R., Chen Y.-H., Chang H.-C., Chan R.-C., Wei S.-H., Wang R.-Y. (2015). Effects of interactive visual feedback training on post-stroke pusher syndrome: A pilot randomized controlled study. *Clinical Rehabilitation*.

[23] In T., Lee K., Song C. (2016). Virtual reality reflection therapy improves balance and gait in patients with chronic stroke: Randomized controlled trials. *Medical Science Monitor*.

[24] Hoermann S., Franz E. A., Regenbrecht H., Fridman E. A. (2012). Referred Sensations Elicited by Video-Mediated Mirroring of Hands. *PLoS ONE*.

[25] Lum P. S., Burgar C. G., Van Der Loos M., Shor P. C., Majmundar M., Yap R. (2006). MIME robotic device for upper-limb neurorehabilitation in subacute stroke subjects: a follow-up study. *Journal of Rehabilitation Research and Development *.

[26] Burgar C. G., Lum P. S., Erika Scremin A. M. (2011). Robot-assisted upper-limb therapy in acute rehabilitation setting following stroke: department of veterans affairs multisite clinical trial. *Journal of Rehabilitation Research and Development *.

[27] Kang J., Chun M. H., Choi S. J., Chang M. C., Yi Y. G. (2017). Effects of mirror therapy using a tablet PC on central facial paresis in stroke patients. *Annals of Rehabilitation Medicine*.

[28] Merians A. S., Tunik E., Fluet G. G., Qiu Q., Adamovich S. V. (2009). Innovative approaches to the rehabilitation of upper extremity hemiparesis using virtual environments. *European Journal of Physical and Rehabilitation Medicine*.

[29] Hoermann S., Hale L., Winser S., Regenbrecht H. (2012). *Augmented Reflection Technology for Stroke Rehabilitation – A Clinical Feasibility Study*.

[30] Penelle B., Mouraux D., Brassinne E., Tuna T., Nonclercq A., Warzée N. 3D augmented reality applied to the treatment of neuropathic pain.

[31] Mehnert J., Brunetti M., Steinbrink J., Niedeggen M., Dohle C. (2013). Effect of a mirror-like illusion on activation in the precuneus assessed with functional near-infrared spectroscopy. *Journal of Biomedical Optics*.

[32] Lee H., Li P., Fan S. (2015). Delayed mirror visual feedback presented using a novel mirror therapy system enhances cortical activation in healthy adults. *Journal of NeuroEngineering and Rehabilitation*.

[33] Peterzell D. H., Kennedy J. F. (2016). Psychophysical investigations into Ramachandran’s mirror visual feedback for phantom limb pain: video-based variants for unilateral and bilateral amputees, and temporal dynamics of paresthesias. *Electronic Imaging*.

[34] Jaewon Beom (2016). *A 2-Axis Robotic Mirror Therapy System to Enhance Proprioception and Functional Recovery of Hemiplegic Arms in Patients with Stroke*.

[35] Emerson I., Potgieter J., Xu W. Evaluation of a prototype integrated robotic and virtual mirror therapy system for stroke rehabilitation.

[36] Kim W., Beom J., Park C. (2018). Reliability and Validity of Attitude and Heading Reference System Motion Estimation in a Novel Mirror Therapy System. *Journal of Medical and Biological Engineering*.

[37] Murray C. D., Pettifer S., Howard T. (2007). The treatment of phantom limb pain using immersive virtual reality: three case studies. *Disabil Rehabil*.

[38] Cole J., Crowle S., Austwick G., Henderson Slater D. (2009). Exploratory findings with virtual reality for phantom limb pain; From stump motion to agency and analgesia. *Disability and Rehabilitation*.

[39] Mercier C., Sirigu A. (2009). Training with virtual visual feedback to alleviate phantom limb pain. *Neurorehabilitation and Neural Repair*.

[40] Sato K., Fukumori S., Matsusaki T. (2010). Nonimmersive virtual reality mirror visual feedback therapy and its application for the treatment of complex regional pain syndrome: An open-label pilot study. *Pain Medicine*.

[41] Alphonso A. L., Monson B. T., Zeher M. J. (2012). Use of a virtual integrated environment in prosthetic limb development and phantom limb pain. *Studies in Health Technnologies and Informatics*.

[42] Shiri S., Feintuch U., Lorber-Haddad A. (2012). Novel virtual reality system integrating online self-face viewing and mirror visual feedback for stroke rehabilitation: Rationale and feasibility. *Topics in Stroke Rehabilitation*.

[43] Won A. S., Collins T. A. (2012). Non-Immersive, Virtual Reality Mirror Visual Feedback for Treatment of Persistent Idiopathic Facial Pain. *Pain Medicine*.

[44] González D. S., Castellini C. (2013). A realistic implementation of ultrasound imaging as a human-machine interface for upper-limb amputees. *Frontiers in Neurorobotics*.

[45] Barton G. J., De Asha A. R., Van Loon E. C. P., Geijtenbeek T., Robinson M. A. (2014). Manipulation of visual biofeedback during gait with a time delayed adaptive Virtual Mirror Box. *Journal of NeuroEngineering and Rehabilitation*.

[46] Ortiz-Catalan M., Sander N., Kristoffersen M. B., Håkansson B., Brånemark R. (2014). Treatment of phantom limb pain (PLP) based on augmented reality and gaming controlled by myoelectric pattern recognition: A case study of a chronic PLP patient. *Frontiers in Neuroscience*.

[47] Schuster-Amft C., Henneke A., Hartog-Keisker B. (2015). Intensive virtual reality-based training for upper limb motor function in chronic stroke: a feasibility study using a single case experimental design and fMRI. *Disability and Rehabilitation: Assistive Technology*.

[48] Diers M., Kamping S., Kirsch P. (2015). Illusion-related brain activations: A new virtual reality mirror box system for use during functional magnetic resonance imaging. *Brain Research*.

[49] Beom J., Koh S., Nam H. S. (2016). Robotic Mirror Therapy System for Functional Recovery of Hemiplegic Arms. *Journal of Visualized Experiments*.

[50] Mouraux D., Brassinne E., Sobczak S. (2016). 3D augmented reality mirror visual feedback therapy applied to the treatment of persistent, unilateral upper extremity neuropathic pain: a preliminary study. *Journal of Manual & Manipulative Therapy*.

[51] Chau B., Phelan I., Ta P., Humbert S., Hata J., Tran D. (2017). Virtual reality therapy with myoelectric control for treatment-resistant phantom limb pain: case report. *Innovations in Clinical Neurosciences*.

[52] Dunn J., Yeo E., Moghaddampour P., Chau B., Humbert S. (2017). Virtual and augmented reality in the treatment of phantom limb pain: A literature review. *NeuroRehabilitation*.

[53] Harvie D. S., Smith R. T., Hunter E. V., Davis M. G., Sterling M., Lorimer Moseley G. (2017). Using visuo-kinetic virtual reality to induce illusory spinal movement: The MoOVi illusion. *PeerJ*.

[54] Kim J., Kim J. Robot-assisted mirroring exercise as a physical therapy for hemiparesis rehabilitation.

[55] Marghi Y. M., Farjadian A. B., Yen S., Erdogmus D. EEG-guided robotic mirror therapy system for lower limb rehabilitation.

[56] Nam H. S., Koh S., Beom J. (2017). Recovery of proprioception in the upper extremity by robotic mirror therapy: A clinical pilot study for proof of concept. *Journal of Korean Medical Science*.

[57] Yarossi M., Manuweera T., Adamovich S. V., Tunik E. (2017). The effects of mirror feedback during target directed movements on ipsilateral corticospinal excitability. *Frontiers in Human Neuroscience*.

[58] Ambron E., Miller A., Kuchenbecker K. J., Buxbaum L. J., Coslett H. B. (2018). Immersive low-cost virtual reality treatment for phantom limb pain: Evidence from two cases. *Frontiers in Neurology*.

[59] Fuentes M. A., Borrego A., Latorre J. (2018). Combined Transcranial Direct Current Stimulation and Virtual Reality-Based Paradigm for Upper Limb Rehabilitation in Individuals with Restricted Movements. A Feasibility Study with a Chronic Stroke Survivor with Severe Hemiparesis. *Journal of Medical Systems*.

[60] Bae J.-H., Kim Y.-M., Moon I. Wearable hand rehabilitation robot capable of hand function assistance in stroke survivors.

[61] Oouchida Y., Izumi S.-I. Imitation movement reduces the phantom limb pain caused by the abnormality of body schema.

[62] Rinderknecht M. D., Kim Y., Santos-Carreras L., Bleuler H., Gassert R. Combined tendon vibration and virtual reality for post-stroke hand rehabilitation.

[63] Saleh S., Adamovich S. V., Tunik E. Visual feedback discordance mediates changes in brain activity and effective connectivity: A stroke fMRI dynamic causal modeling study.

[64] Fukumori S., Gofuku A., Isatake K., Sato K. Mirror thrapy system based virtual reality for chronic pain in home use.

[65] Hoermann S., Santos L. F., Morkisch N. Computerized mirror therapy with augmented reflection technology for stroke rehabilitation: A feasibility study in a rehabilitation center.

[66] Shahbazi M., Atashzar S. F., Tavakoli M., Patel R. V. (2016). Robotics-assisted mirror rehabilitation therapy: A therapist-in-the-loop assist-as-needed architecture. *IEEE/ASME Transactions on Mechatronics*.

[67] Llorens R., Borrego A., Latorre J., Alcaniz M., Colomer C., Noe E. A combined transcranial direct current stimulation and virtual reality-based intervention on upper limb function in chronic stroke survivors with severe hemiparesis.

[68] Su Y., Wu Y., Gao Y., Dong W., Sun Y., Du Z. A upper limb rehabilitation system with motion intention detection.

[69] Swee S. K., You L. Z., Hang B. W., Kiang D. K. Development of rehabilitation system using virtual reality.

[70] Hesse S., Schulte-Tigges G., Konrad M., Bardeleben A., Werner C. (2003). Robot-assisted arm trainer for the passive and active practice of bilateral forearm and wrist movements in hemiparetic subjects. *Archives of Physical Medicine and Rehabilitation*.

[71] Lum P. S., Burgar C. G., Shor P. C. (2004). Evidence for improved muscle activation patterns after retraining of reaching movements with the MIME robotic system in subjects with post-stroke hemiparesis. *IEEE Transactions on Neural Systems and Rehabilitation Engineering*.

[72] Lozano J. A., Montesa J., Juan M. C. (2005). VR-Mirror: A Virtual Reality System for Mental Practice in Post-Stroke Rehabilitation. *International Symposium on Smart Graphics*.

[73] Desmond D. M., Og'Neill K., De Paor A., McDarby G., MacLachlan M. (2006). Augmenting the reality of phantom limbs: Three case studies using an augmented mirror box procedure. *Journal of Prosthetics and Orthotics*.

[74] Murray C. D., Patchick E., Pettifer S. (2006). Investigating the efficacy of a virtual mirror box in treating phantom limb pain in a sample of chronic sufferers. *International Journal on Disability and Human Development*.

[75] Lewis G. N., Perreault E. J. (2009). An assessment of robot-assisted bimanual movements on upper limb motor coordination following stroke. *IEEE Transactions on Neural Systems and Rehabilitation Engineering*.

[76] Kadivar Z., Sung C., Thompson Z., O'Malley M., Liebschner M., Deng Z. (2011). Comparison of reaching kinematics during mirror and parallel robot assisted movements. *Studies in Health Technology and Informatics*.

[77] Regenbrecht H., McGregor G., Ott C. Out of reach? A novel AR interface approach for motor rehabilitation.

[78] Regenbrecht H. T., Franz E. A., McGregor G., Dixon B. G., Hoermann S. (2011). Beyond the looking glass: Fooling the brain with the augmented mirror box. *Presence: Teleoperators and Virtual Environments*.

[79] Barton G. J., Asha A. R. D., Geijtenbeek T., Robinson M. A. (2012). Development of a virtual mirror box for spatial and temporal manipulation of visual feedback on body movement during gait: A technical evaluation. *Gait & Posture*.

[80] Casas X., Herrera G., Coma I., Fernández M. A Kinect-based Augmented Reality system for individuals with autism spectrum disorders.

[81] Liao W.-W., Wu C.-Y., Hsieh Y.-W., Lin K.-C., Chang W.-Y. (2012). Effects of robot-assisted upper limb rehabilitation on daily function and real-world arm activity in patients with chronic stroke: a randomized controlled trial. *Clinical Rehabilitation*.

[82] Regenbrecht H., Hoermann S., McGregor G. (2012). Visual manipulations for motor rehabilitation. *Computers & Graphics*.

[83] Ueki S., Kawasaki H., Ito S. (2012). Development of a hand-assist robot with multi-degrees-of-freedom for rehabilitation therapy. *IEEE/ASME Transactions on Mechatronics*.

[84] Perry B. N., Alphonso A. L., Tsao J. W., Pasquina P. F., Armiger R. S., Moran C. W. A Virtual Integrated Environment for phantom limb pain treatment and Modular Prosthetic Limb training.

[85] Trojan J., Diers M., Fuchs X. (2013). An augmented reality home-training system based on the mirror training and imagery approach. *Behavior Research Methods*.

[86] Wake N., Sano Y., Oya R., Sumitani M., Kumagaya S.-I., Kuniyoshi Y. Multimodal virtual reality platform for the rehabilitation of phantom limb pain.

[87] Sano Y., Wake N., Ichinose A. (2016). Tactile feedback for relief of deafferentation pain using virtual reality system: A pilot study. *Journal of NeuroEngineering and Rehabilitation*.

[88] Ichinose A., Sano Y., Osumi M., Sumitani M., Kumagaya S.-I., Kuniyoshi Y. (2017). Somatosensory feedback to the cheek during virtual visual feedback therapy enhances pain alleviation for phantom arms. *Neurorehabilitation and Neural Repair*.

[89] Osumi M., Ichinose A., Sumitani M. (2017). Restoring movement representation and alleviating phantom limb pain through short-term neurorehabilitation with a virtual reality system. *European Journal of Pain*.

[90] Pozeg P., Palluel E., Ronchi R. (2017). Virtual reality improves embodiment and neuropathic pain caused by spinal cord injury. *Neurology*.

[91] O'Neill K., Maclachlan M., Mcdarby G. (Mai 2018). *An Investigation into the performance of a Virtual Mirror Box for the treatment of Phantom Limb Pain in Amputees using Augmented Reality Technology*.

[92] Amano T., González-Varo J. P., Sutherland W. J. (2016). Languages are still a major barrier to global science. *PLoS Biology*.

[93] Sayre J. W., Toklu H. Z., Ye F., Mazza J., Yale S. (2017). Case reports, case series – from clinical practice to evidence-based medicine in graduate medical education. *Cureus*.

[94] McCambridge J., Witton J., Elbourne D. R. (2014). Systematic review of the Hawthorne effect: new concepts are needed to study research participation effects. *Journal of Clinical Epidemiology*.

[95] Bouchet C., Guillemin F., Briançon S. (1996). Nonspecific effects in longitudinal studies: Impact on quality of life measures. *Journal of Clinical Epidemiology*.

[96] Iles R. L. (1997). *Guidebook to Better Medical Writing*.

[97] Pälmke M., Von Piekartz H., Zalpour C., Schüler T., Morisse K. A new perspective for Virtual Mirror Therapy: Developing a low-cost-high-convenient environment utilising the Wiimote.

[98] Sanson-Fisher R. W., Bonevski B., Green L. W., D'Este C. (2007). Limitations of the randomized controlled trial in evaluating population-based health interventions. *American Journal of Preventive Medicine*.

[99] Borenstein M., Hedges L. V., Higgins J. P. T., Rothstein H. R. (2009). Criticisms of meta-analysis. *Introduction to Meta-Analysis*.

[100] Batson S., Webb N., Greenall G. (2015). Meta-analysis to support technology submissions to health technology assessment authorities: criticisms by nice and evidence review groups in the Uk. *Value in Health*.

[101] Laver K. E., Lange B., George S., Deutsch J. E., Saposnik G., Crotty M. (2017). Virtual reality for stroke rehabilitation. *Cochrane Database of Systematic Reviews*.

[102] Mehrholz J., Pohl M., Platz T., Kugler J., Elsner B. (2015). Electromechanical and robot‐assisted arm training for improving activities of daily living, arm function, and arm muscle strength after stroke. *The Cochrane Library*.

[103] Mehrholz J., Thomas S., Werner C., Kugler J., Pohl M., Elsner B. (2017). Electromechanical‐assisted training for walking after stroke. *The Cochrane Library*.

[104] Mehrholz J., Hädrich A., Platz T., Kugler J., Pohl M. (2012). Electromechanical and robot-assisted arm training for improving generic activities of daily living, arm function, and arm muscle strength after stroke. *Cochrane Database of Systematic Reviews*.

[105] Kwakkel G., Kollen B. J., Krebs H. I. (2008). Effects of robot-assisted therapy on upper limb recovery after stroke: a systematic review. *Neurorehabilitation and Neural Repair*.

[106] Mehrholz J., Kugler J., Pohl M. (2012). Locomotor training for walking after spinal cord injury. *The Cochrane Library*.

[107] Bujar M., Donelan R., McAuslane N., Walker S., Salek S. (2017). Assessing the quality of decision making in the development and regulatory review of medicines: Identifying biases and best practices. *Therapeutic Innovation and Regulatory Science*.

[108] Bujar M., McAuslane N., Walker S. R., Salek S. (2017). Evaluating quality of decision-making processes in medicines’ development, regulatory review, and health technology assessment: a systematic review of the literature. *Frontiers in Pharmacology*.

[109] Gøtzsche P. C. (2011). Why we need easy access to all data from all clinical trials and how to accomplish it. *Trials*.

[110] Lundh A., Lexchin J., Mintzes B., Schroll J. B., Bero L., The Cochrane Collaboration (2017). Industry sponsorship and research outcome. *Cochrane Database of Systematic Reviews*.

[111] Patel S. V., Yu D., Elsolh B., Goldacre B. M., Nash G. M. (2017). Assessment of conflicts of interest in robotic surgical studies: validating author*ʼ*s declarations with the open payments database. *Annals of Surgery*.

