# Supplementary material for: Do Robotics and Virtual Reality Add Real Progress to Mirror Therapy Rehabilitation? A Scoping Review
Source: Rehabil Res Pract. 2018 Aug 19;2018:6412318. doi: 10.1155/2018/6412318 (PMC6120256; doi:10.1155/2018/6412318)
Supplement: Supplementary Materials — Table S1. Exhaustive list of rationales cited in the studies for conducting research on mirror robotic systems or mirror virtual reality rather than standard mirror therapy. Table S2. Exhaustive list of outcomes nature used in the included studies. Table S3. Exhaustive list of names used in the included studies to designate the different types of second-generation mirror therapy device. [file 6412318.f1.pdf]

Table S1 : Exhaustive list of rationals cited in the studies for conducting research on mirror robotic systems or mirror virtual reality rather than standard mirror therapy

**Rational**	**Number of studies**
Multisensory feedback (to facilitate neuroplasticity)	19
To increase the range and difficulty of possible training task	15
Increasing motivation and engagment	11
Intensive and repetitive training	9
Customizable environments	9
To accomplish different bimanual coordination movements	7
Faster or greater recovery	7
To move the affected arm with severe motor deficits	6
Can work for patients with bilateral amputations or paralyses	5
To operate within a wide spatial dimension	5
To create a complete illusion	5
Cost-reduction in healthcare (decreasing reimbursable patient-therapist contact time)	4
Providing more lifelike visual cues	3
To beter understand the cortical mechanisms during mirror therapy	3
Being able to cross the hands in the visual display so that the left hand appears on the right side and vice versa	3
For patients who are not responding adequately to standard mirror terapy	3
To quantify therapy and assessment procedures	2
To active participation of a patient	2
To develop a strong sens of ownership of the displayed limb	2
To reduce workload of therapists	1
Easy to use	1
To maintain correct balance (for lower limb rehabilitation)	1
To produce less tension in the cervical posture	1
Making mirror therapy possible for facial paralysis	1
Rapid pace of development in this field	1
For use during functional magnetic resonance imaging	1
To treat multiple patients simultaneously	1
To perform rehabilitation at home	1
Feasible	1
Robotic orthesis function	1
To independently carry out rehabilitation exercises	1

Table S2 : Exhaustive list of outcomes nature used in the included studies

**Outcomes nature**	**Number of studies**
Pain	25
Satisfaction in the system	18
Body functions and activities	17
Motor assessment	10
Spasticity level	9
Illusion intensity	8
fMRI data	7
Muscle strenght	5
Electromyography measurements	5
Operability of movements	4
Gait	4
EEG measurements	4
Usability of the system	3
Performance of the device	3
Neurological status	3
Hemineglect	3
Balance	3
Accuracy of the motion estimation system	3
Time upon completion of the pick and place training	2
Sense of agency	2
Safesty of the system	2
Proprioception	2
Patients' own embodied experience	2
Movement and force trajectoire	2
Medication times	2
Handedness	2
Hand identification	2
Feasibility	2
Experienced ownership	2
Detecting the user’s motion intentions	2
Adequate patient performance	1
Changes in prosthetic hardware	1
Degree of facial paresis	1
Functional near-infrared spectroscopy	1
Motor coordination	1
Perceived head orientation	1
Performance	1
Quality of life	1
Referred sensations	1
Self-confidence	1
Self-motion control	1
Sense of delay	1
Severity of Pusher syndrom	1
Task kinematics	1
Temperature judgment	1
Vividness	1

Table S3 : Exhaustive list of names used in the included studies to designate the different types of second generation mirror therapy device.

**Names**	**Number of studies**
(intensive/immersive/low-cost) Virtual reality (interventions/ based paradigm/system/environment/ based training/mirror box system/programs/technology/treatment/therapy/workbench)	37
(Mirror) visual feedback (therapy)	10
(3D) augmented reality (mirror) (system/environment)	9
Augmented reality (system/technology)	8
Virtual (reality) mirror (box/paradigm)	7
(mirror/virtual) Visual (bio)feedback (training program/therapy/discordance)	5
Robot(ic)-assisted (mirroring exercise) (mirror) (rehabilitation/therapy/treatment)	5
Robotic therapy/device/system/trainer	5
Robot-aided (or assisted) mirror therapy	4
Robotic mirror therapy (system)	4
Augmented reflection technology (system)	3
Mirror (and parallel)robot (assisted) system/movements	3
Robotic rehabilitation (system)	3
Augmented mirror (box)	2
Computerised mirror therapy	2
Mirror(ed) feedback	2
Novel mirror therapy system	2
Rehabilitation robot(ic) (system)	2
Robotic devices	2
Video-mediated (mirroring)	2
(full) Body illusions	1
Assistive robot for mirror therapy	1
Augmented environments	1
Augmented mirror box procedure	1
Based on mirror training	1
Brain-computer interface	1
Computer mediated visual feedback	1
Computer-generated interactive	1
Digital mirror therapy	1
Haptic device design	1
Head-mounted display	1
Human-centered computing	1
Human-computer interaction	1
Human-machine interface/interactions	1
Human-robotic intervention	1
Interaction devices	1
Low-cost immersive virtual reality	1

Mirror therapy system based virtual reality	1
Mirror therapy using a tablet PC	1
Mirror-like illusion	1
Mixed reality rehabilitation system	1
Motion assistance robot	1
Motion estimation system	1
Real-time video	1
Robotic and Virtual Mirror Therapy System	1
Robotic exoskeleton	1
Simple video feedback	1
Ultrasound imaging	1
Video-based variants	1
Virtual and augmented reality (mirror therapy treatments)	1
Virtual body	1
Virtual environments	1
Virtual integrated environment	1
Virtual leg illusion	1
Virtual rehabilitation devices	1
(Virtual) (neurorehabilative) visual feedback therapy	1
Visuo-kinetic virtual reality	1
